# Inability of Low Oxygen Tension to Induce Chondrogenesis in Human Infrapatellar Fat Pad Mesenchymal Stem Cells

**DOI:** 10.3389/fcell.2021.703038

**Published:** 2021-07-26

**Authors:** Samia Rahman, Alexander R. A. Szojka, Yan Liang, Melanie Kunze, Victoria Goncalves, Aillette Mulet-Sierra, Nadr M. Jomha, Adetola B. Adesida

**Affiliations:** ^1^Laboratory of Stem Cell Biology and Orthopedic Tissue Engineering, Division of Orthopedic Surgery and Surgical Research, Department of Surgery, University of Alberta, Edmonton, AB, Canada; ^2^Division of Otolaryngology-Head and Neck Surgery, Department of Surgery, University of Alberta Hospital, Edmonton, AB, Canada

**Keywords:** chondrogenesis, adipose tissue, human mesenchymal stem cells, oxygen tension, xenograft, *in vivo* calcification

## Abstract

**Objective:**

Articular cartilage of the knee joint is avascular, exists under a low oxygen tension microenvironment, and does not self-heal when injured. Human infrapatellar fat pad-sourced mesenchymal stem cells (IFP-MSC) are an arthroscopically accessible source of mesenchymal stem cells (MSC) for the repair of articular cartilage defects. Human IFP-MSC exists physiologically under a low oxygen tension (i.e., 1–5%) microenvironment. Human bone marrow mesenchymal stem cells (BM-MSC) exist physiologically within a similar range of oxygen tension. A low oxygen tension of 2% spontaneously induced chondrogenesis in micromass pellets of human BM-MSC. However, this is yet to be demonstrated in human IFP-MSC or other adipose tissue-sourced MSC. In this study, we explored the potential of low oxygen tension at 2% to drive the *in vitro* chondrogenesis of IFP-MSC. We hypothesized that 2% O_2_ will induce stable chondrogenesis in human IFP-MSC without the risk of undergoing endochondral ossification at ectopic sites of implantation.

**Methods:**

Micromass pellets of human IFP-MSC were cultured under 2% O_2_ or 21% O_2_ (normal atmosphere O_2_) in the presence or absence of chondrogenic medium with transforming growth factor-β3 (TGFβ3) for 3 weeks. Following *in vitro* chondrogenesis, the resulting pellets were implanted in immunodeficient athymic nude mice for 3 weeks.

**Results:**

A low oxygen tension of 2% was unable to induce chondrogenesis in human IFP-MSC. In contrast, chondrogenic medium with TGFβ3 induced *in vitro* chondrogenesis. All pellets were devoid of any evidence of undergoing endochondral ossification after subcutaneous implantation in athymic mice.

## Introduction

Articular cartilage is avascular and exists under a low oxygen tension microenvironment (Lund-Olesen, 1970; Brighton and Heppenstall, 1971a, b). Articular cartilage injuries do not heal spontaneously due to its avascularity and the low mitotic activity of its resident articular chondrocytes (Martin and Buckwalter, 2003). If the injuries are left untreated, they become a risk factor for the early development of osteoarthritis (Muthuri et al., 2011). To this end, a variety of cell-based approaches including autologous chondrocyte implantation and multipotent mesenchymal stem cells (MSC) have been assessed to augment articular cartilage healing and mitigate the onset of osteoarthritis, in particular, knee osteoarthritis (Brittberg et al., 1994, 2001; Peterson et al., 2000; Hwang et al., 2011; Bornes et al., 2014, 2018; Mobasheri et al., 2014; Fellows et al., 2016).

The abundance of MSC in adipose tissue and their ease of accessibility have made adipose-derived MSC a cell source of significant interest for articular cartilage repair (Zuk et al., 2001, 2002; Huang et al., 2004; Fellows et al., 2016; Freitag et al., 2020). Although several studies have demonstrated the *in vitro* chondrogenesis of adipose-derived MSC with growth factors (i.e., TGFβ1, TGFβ3, and BMP-6) in the absence or presence of low oxygen tension, no study has investigated the potential of low oxygen tension alone to drive the *in vitro* chondrogenesis of adipose-derived MSC (Zuk et al., 2001; Erickson et al., 2002; Awad et al., 2003; Huang et al., 2004; Wang et al., 2005; Estes et al., 2006; Afizah et al., 2007; Hennig et al., 2007; Khan et al., 2007; Malladi et al., 2007; Mehlhorn et al., 2007; Cheng et al., 2009; Kim and Im, 2009; Martinez-Lorenzo et al., 2009; Diekman et al., 2010; Merceron et al., 2010).

Xu et al. (2007) demonstrated that expansion of adipose-derived MSC under 2% O_2_ prior to *in vitro* chondrogenic differentiation in three-dimensional micromass culture under 21% O_2_ enhanced early chondrogenic differentiation. Xu et al. (2007) concluded that the pre-expansion culture of the adipose MSC under the 2% O_2_ facilitated the selection of chondroprogenitors. More recently, Marsano et al. (2016) demonstrated the spontaneous *in vitro* chondrogenesis of bone marrow-derived MSC under a low oxygen tension of 2% O_2_. Interestingly, both bone marrow- and adipose-derived MSCs physiologically exist in a low oxygen tension microenvironment (Ceradini et al., 2004; Choi et al., 2014). Moreover, during embryonic development, cartilage formation is preceded by MSC condensation in an avascular microenvironment with gross hypoxia and hypoxia-inducible factor 1 alpha (HIF-1α)-mediated signaling involvement (Robins et al., 2005; Provot and Schipani, 2007).

Thus, we ask the question: can low oxygen tension alone drive *in vitro* chondrogenesis of human adipose-derived MSCs? And will the chondrogenic outcome be hypertrophic in nature with the risk of undergoing endochondral ossification after subcutaneous implantation in an immunodeficient mouse model, as previously demonstrated with chondrogenically differentiated bone marrow MSC (Pelttari et al., 2006)?

In this study, we investigated the chondroinductive potential of low oxygen tension at 2% in human knee infrapatellar fat pad MSC (IFP-MSC). It is noteworthy that *in vitro* IFP-MSC has been reported to show stronger chondrogenic capacity than bone marrow-, subcutaneous adipose-, and umbilical cord-derived MSC (Ding et al., 2015). Moreover, further stratification of IFP-MSC into perivascular (i.e., pericytes) and non-perivascular (i.e., adventitial cells) subpopulations have revealed the superior chondrogenic capacity of the perivascular fraction (Hindle et al., 2017).

We hypothesized that low oxygen tension or hypoxia (HYP, 2% O_2_) would induce non-hypertrophic chondrogenesis. Additionally, we hypothesized that the hypoxic culture conditions would produce superior chondrogenesis relative to intermittent reoxygenation (Re-Ox).

## Materials and Methods

### Ethics and Sample Collection

Infrapatellar fat pad (IFP) specimens with non-identifying information of donors were collected from the University of Alberta Hospital. The University of Alberta’s Health Research Ethics Board-Biomedical Panel (Study ID: Pro00018778) waived the need for written informed consent of the donors, as specimens were intended to be discarded in the normal course of the surgical procedures. All animal use and associated procedures were approved and undertaken under the direction and approval of the University of Alberta Animal Care and Use Committee (AUP 0001363).

### Isolation and Expansion of Human Infrapatellar Fat Pad Mesenchymal Stem Cells

Tissue from the IFP was collected from male donors ages 21–59 (Table 1) who had experienced acute knee injuries and did not have osteoarthritis. The basic information of the donors is summarized in Table 1. IFP samples were first filtered through a sterile 100-μm cell strainer. We then treated the samples with type II collagenase (0.15% w/v; 300 U/mg solid; Worthington, Lakewood, NJ, United States) in phosphate-buffered saline (PBS) (Sigma-Aldrich, St. Louis, MO, United States) for 1 h at 37°C to release the cells. Next, we filtered the digested tissue using a sterile 100-μm cell strainer (Falcon, BD Biosciences, Franklin Lakes, NJ, United States) to obtain the cell suspension. We neutralized the collagenase in the cell suspension by adding alpha-minimum essential medium (α-MEM) supplemented with 10% v/v heat-inactivated fetal bovine serum (FBS), 100 mM 4-(2-hydroxyethyl)-1-piperazineethanesulfonic acid (HEPES), 1 mM sodium pyruvate (all from Sigma-Aldrich Co.), 100 U/ml penicillin, 100 μg/ml streptomycin, and 0.29 mg/ml glutamine (PSG; Life Technologies, Burlington, ON, Canada). Cells were then isolated by 10 min of centrifugation at 1,500 rpm and then resuspended in PBS. The number of mononucleated cells (MNCs) was then counted first by crystal violet (Sigma-Aldrich) nuclei staining and then by using a hemocytometer.

**TABLE 1:** IFP-MSC donor information along with cumulative population doubling (CPD).

Donor	Gender	Age	BMI (kg/m^2^)	Cumulative population doublings at P2
1	M	59	21.5	13.3
2	M	50	45.2	15.6
3	M	30	32.2	13.63
4	M	36	31.6	14.4
5	M	25	23.9	13.0
6	M	47	26.7	13.8
7	M	21	22.7	16.17

The isolated nucleated cells were then expanded in one of two ways, using the medium described above with 5 ng/ml of fibroblast growth factor-2 (FGF-2) (Neuromics, Minneapolis, MN, United States, Catalog#: PR80001) to maintain the chondrogenic potential of the cells, in normoxia (21% O_2_; NRX) at 37°C in a humidified incubator with 5% CO_2_. Method 1: cells were either plated in culture flasks with 125,000 cells per 75 cm^2^ (donors: 5, 6, 7) at initial seeding and then flasks were duplicated at each passage (i.e., the cells of two flasks would be set up in four flasks and allowed to grow). Method 2: the cells were replated after each passage to have 5,000 cells per cm^2^ (donors: 1, 2, 3, 4). The nucleated cells grew and adhered for 7 days before the first medium change. After this, the medium was changed twice each week, until the cells were 80% confluent. The adherent IFP-MSC were then detached using 0.05% w/v trypsin-EDTA (Corning, Mediatech, Inc., Manassas, VA, United States) and expanded in normoxia until passage 2 as previously described (Weiss et al., 2017). To achieve this, we first expanded the cells in normoxia (21% O_2_) and then cultured them in hypoxia (2% O_2_); the choice to expand them in normoxia initially is to be able to see the effects due more clearly to the culture conditions of hypoxia.

### Colony-Forming Unit Fibroblastic Assay

We performed a colony-forming unit fibroblastic assay to ascertain the clonogenic and population doubling characteristics of the IFP-MSC. We plated 500 nucleated cells each in three 100-mm sterile Petri dishes (Becton Dickinson, Mississauga, ON, Canada) and cultured them in NRX with α-MEM supplemented with 10% v/v heat-inactivated FBS, PSG, HEPES, sodium pyruvate, and 5 ng/ml FGF-2 (as above). After 1 week, the non-adherent population was removed by aspiration and the medium was changed twice each week. The culture time used for each IFP-MSC donor was the time needed to reach 80% confluence at P0 and subsequent detachment and splitting to P1 for expansion. The cell colonies were fixed with 10% w/v buffered formalin (3.8% w/v formaldehyde, Anachemia Canada Co., Montreal, QC, Canada), rinsed with PBS (Sigma-Aldrich), and stained with 0.25% w/v crystal violet. We then recorded the number of colonies and used the number of colonies to determine the cell population doubling (CPD; Table 1) of IFP-MSC as described by Solchaga et al. (2010).

### *In vitro* Chondrogenic Differentiation

To test the effects of oxygen and growth factor supplementation on chondrogenic differentiation, we utilized a three-dimensional pellet culture model. After completing passage 2, 0.5 million cells of IFP-MSC were centrifuged for 7 min at 1,500 rpm to make pellets in 1.5 ml sterile conical microtubes (Bio Basic Inc., Markham, ON, Canada.). The pellets were subjected to one of two conditions for the medium (0.5 ml per pellet) during the culture period: with or without TGFβ3 supplementation.

Within each of these two conditions for the medium, the pellets were either cultured in NRX (∼21% O_2_), HYP (2% O_2_), or Re-Ox (2% with intermittent exposure to ∼21% O_2_). During both culture and media changes, the NRX pellets were constantly exposed to ∼21% oxygen tension, and HYP pellets were constantly exposed to 2% oxygen tension in an X3 Xvivo hypoxia workstation (BioSpherix, Parish, NY, United States). The Re-Ox pellets were cultured in 2% oxygen tension but were removed from the hypoxia workstation during media changes and exposed to ∼21% oxygen tension twice per week for 15 min. Pellets in the HYP group were changed with media equilibrated to 2% O_2_. Pellets in the Re-Ox and NRX groups were changed with media containing 21% O_2_. All pellets were cultured for 21 days at 37°C in a humidified incubator with 5% CO_2_, with media changes twice per week (3–4 days apart).

The serum-free media consisted of high glucose DMEM (Sigma-Aldrich) containing 100 units/ml penicillin, 100 μg/ml streptomycin, 2 mM L-glutamine, 10 mM HEPES (Sigma-Aldrich) (all others from Life Technologies), ITS + 1 premix (Corning, Discovery Labware, Inc., Bedford, MA, United States), 100 nM dexamethasone, 365 μg/ml ascorbic acid 2-phosphate, 125 μg/ml human serum albumin, and 40 μg/ml L-proline (all from Sigma-Aldrich). The TGFβ3-supplemented condition had 10 ng/ml transforming growth factor β3 (TGFβ3; ProSpec, East Brunswick, NJ, United States, Catalog#: cyt-113) added to the serum-free media, while the group without TGFβ3 supplementation did not have this added to the media.

For each of the six conditions (± TGFβ3 supplementation and NRX/HYP/Re-Ox), two pellets were set up for each donor for biochemical analysis [glycosaminoglycan (GAG) and DNA analysis], histological analysis, and qPCR. For three (1, 2, 4) of the seven donors, two pellets were set up for each condition for subcutaneous implantation. At the start of the experiment, 0.5 million cells for each donor were suspended in 1 ml of TRIzol (Life Technologies) as a control for monolayer culture gene expression. Medium changes on the pellets were performed twice per week with the same type of serum-free media they were initially cultured in. After 21 days, the pellets were assessed using biochemistry for GAG and DNA content, histology and immunofluorescence for cartilage-specific matrix proteins, and real-time quantitative reverse transcription polymerase chain reaction (qPCR) for gene expression analysis.

### Biochemical Analysis for IFP-MSC Chondrogenesis

After chondrogenic culture for 21 days, the pellets were rinsed with 500 μl of phosphate buffered saline (Sigma-Aldrich). The pellets were then digested overnight at 56°C with 250 μl of proteinase K (1 mg/ml in 50 mM Tris with 1 mM EDTA, 1 mM iodoacetamide, and 10 mg/ml pepstatin A; all from Sigma-Aldrich). The GAG content was measured using a spectrophotometer after 1,9-dimethylmethylene blue binding, with chondroitin sulfate used as standard (Sigma-Aldrich). To determine the DNA content, we used the CyQuant cell proliferation assay kit (Invitrogen, Burlington, ON, Canada) with supplied bacteriophage λ DNA as standard.

### Histology for IFP-MSC Chondrogenesis

Pellets were taken after chondrogenic culture for 21 days or after chondrogenic culture and subcutaneous implantation for 3 weeks and fixed overnight in 10% (v/v) neutral buffered formalin at 4°C, then put in PBS. The pellets were then dehydrated by serially dipping into ethanol baths of increasing concentration and embedded in paraffin wax. These were then cut into 5-μm-thick sections and stained with 0.01% (w/v) Safranin O and counterstained with 0.02% (w/v) fast green (Sigma-Aldrich) with standard methods to reveal proteoglycan matrix deposition.

### Immunofluorescence for IFP-MSC Chondrogenesis

Thick sections at 5 μm of paraffin-embedded pellets were mounted on VWR Microslides Superfrost glass slides (cat# 48311-703), then deparaffinized and rehydrated. The sections for type I, II, and X collagen were next treated with protease XXV (AP-9006-005, Thermo Scientific, Waltham, MA, United States), rinsed with PBS (Sigma-Aldrich), treated with hyaluronidase (H6254, Sigma-Aldrich), and then rinsed with PBS. Samples for collagen X were then treated with 0.2% Triton for 10 min. All samples were blocked for 30 min in 5% (w/v) bovine serum albumin (9998S, Cell Signaling Technology, Danvers, MA, United States) in PBS. For collagen I detection, sections were incubated with primary antibody:rabbit anti-collagen I (CL50111AP-1, Cedarlane, Burlington, ON, United States) (1:200 dilution); for collagen II detection, sections were incubated with primary antibody:mouse anti-collagen II (II-II6B3, Developmental Studies Hybridoma Bank, Iowa City, IA, United States) (1:200 dilution); and for collagen X detection, sections were incubated with primary antibody:rabbit anti-collagen X (58632, Abcam, Cambridge, United Kingdom) using a 1:100 dilution in 4°C overnight. This was followed by incubating collagen I and X slides with goat anti-mouse IgG (H&L Alexa Fluor 488, Abcam) (1:200 dilution) and collagen II slides with goat anti-rabbit IgG (H&L Alexa Fluor 594, Abcam) (1:200 dilution) for 30 min. Sections were then stained with DAPI (4′,6-diamidino-2-phenylindole, Cedarlane) and mounted with glycerol:PBS (1:1). The slides were visualized by an Eclipse Ti-S microscope (Nikon Canada, Mississauga, Canada).

### Gene Expression Analysis

For pellets that were cultured with TGFβ3 supplementation, we ground the pellets with Molecular Grinding Resin (G-Biosciences, Saint Louis, MO, United States, 786-138PR) extracted total RNA using Tri-Reagent (Sigma-Aldrich). Due to the considerably smaller size of the pellets cultured without TGFβ3 supplementation, we ground the pellets using the same grinding resin as above and used the RNeasy mini kit (Qiagen, Toronto, ON, Canada); 100 ng of the isolated total RNA was reverse transcribed into cDNA for qPCR analysis using the gene-specific primers listed in Supplementary Table 1. Expression of genes of interest was normalized to the mean expression level of reference genes *YWHAZ*, β*-actin*, and *B2M* (Foldager et al., 2009; Munir et al., 2014) and presented using the 2ΔCt method (Schmittgen and Livak, 2008).

### Subcutaneous Implantation in Nude Mice

To evaluate the phenotypic stability and potential for ossification, we implanted pellets subcutaneously at the back of nude mice for 21 days. Four small incisions were made subcutaneously in the back (two cranial and two caudal) of athymic male nude CD-1 mice (*n* = 5, Charles River, Wilmington, MA, United States). These incisions were closed by sutures and cyanoacrylate tissue adhesive. The pellets were placed in duplicates with pellets from the same donor with the same culture conditions (medium and oxygen condition) together; altogether 20 groups (40 pellets) were implanted. After the 21 days of implantation, the constructs were recovered while the animals were under anesthesia and then euthanized. There were no adverse events recorded with the five mice, and 19 of the 20 groups of implanted pellets were recovered; one group was unable to be found during harvest and was not recovered. After removing the pellets from the animal, we removed excess mouse connective tissue from the pellets using a scalpel. The samples were then fixed in 10% (w/v) formalin for 24 h and paraffin embedded as described above for histology. To reveal proteoglycan matrix deposition, we stained 5-μm-thick sections with 0.01% (w/v) Safranin O and counterstained with 0.02% (w/v) fast green (Sigma-Aldrich). To assess the phenotypic stability, we looked for signs of bone formation by deposition of calcium phosphate mineral *via* Alizarin Red staining (2% w/v, pH 4.2, Sigma-Aldrich).

### Alizarin Red S Staining

Alizarin Red staining was used to visualize calcium phosphate mineral formation in pellets that had been cultured for 21 days and then implanted in a nude mouse for 21 days. The 5-μm-thick sections of paraffin-embedded pellets were deparaffinized by serially dipping into ethanol baths of increasing concentration, then rehydrating with distilled water, and transferred to Alizarin Red S solution (pH 4.2) for 5 min. Sections were then dehydrated with acetone, treated with an acetone:xylene substitute (1:1) solution, cleared in xylene substitute, and mounted.

### Statistical Analysis

A total of seven independent experiments were performed with specimens from seven donors for IFP-MSC. Unless stated specifically, numerical data distribution represents data from these donors each measured at least in independent duplicates and is presented as a bar graph of the mean ± standard deviation. Statistical analyses were performed using SPSS (version 26; IBM Canada Ltd., Markham, ON, Canada), and boxplots were generated using Excel in Microsoft Office 365 ProPlus. Data were tested for normality using the Shapiro–Wilk test, and Levene’s test was used to assess homogeneity of error variances. Statistical differences between the measured parameters of pellets formed. The relationship between measured parameters was determined by Spearman rho correlation coefficient based on confirmation of non-normality of data distribution. Statistically significant differences between multiple groups were assessed by Kruskal–Wallis non-parametric test based on significance of the Levene’s test. Significance was considered when *p* < 0.05.

## Results

### Hypoxia Alone Did Not Induce Chondrogenic Matrix Accumulation in IFP-MSC

**FIGURE 1:**
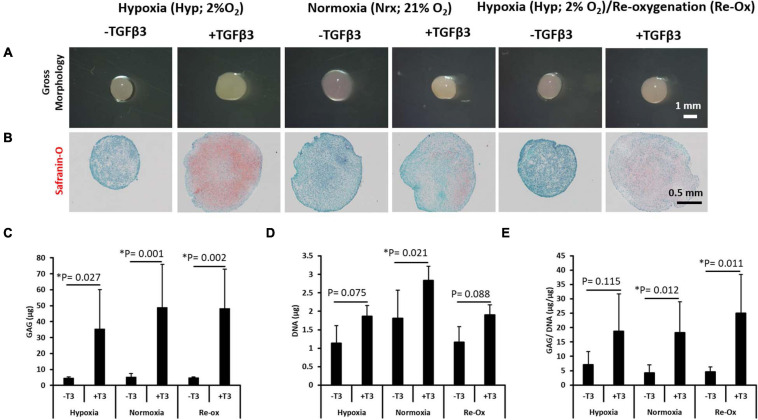
Gross morphology of IFP-MSC pellets after 21 days of *in vitro* culture; Safranin O histochemical staining and biochemical assay for GAG and DNA content. **(A)** Gross morphology of pellets, **(B)** Safranin O staining, **(C)** total GAG content, **(D)** total DNA content, and **(E)** GAG/DNA content. Comparing pellets for seven donors done in duplicates (*N* = 7, *n* = 14 per experimental group) after 21 days of *in vitro* culture under six different conditions. Bar data are mean ± standard deviation. Statistical comparisons were based on Kruskal–Wallis test between without TGFβ3 (i.e., –T3) and with TGFβ3 (i.e., +T3) groups within an oxygen tension group. ^∗^Indicates statistical difference between compared groups with *p*-value < 0.05.

After 3 weeks of culture in chondrogenic media supplemented with and without TGFβ3, pellets were harvested and *in vitro* chondrogenesis was first assessed histologically by Safranin O staining for cartilaginous sulfated GAG extracellular matrix (ECM) detection (Figure 1). All pellets appeared spherical, opaque, smooth, and glistening regardless of TGFβ3 supplementation (Figure 1A). The pellets cultured in the absence of TGFβ3 regardless of the oxygen tension during *in vitro* culture were negative for Safranin O staining (Figure 1B). In contrast, all pellets cultured in the presence of TGFβ3 were positive for Safranin O staining (Figure 1). Moreover, the pellets cultured with TGFβ3 under a constant hypoxia (i.e., 2% oxygen tension) had more intense Safranin O staining (Figure 1B).

In order to quantitate the amount of GAG matrix accumulated in pellets across the different experimental groups, the GAG assay (Farndale et al., 1986) was performed. The GAG amounts measured in all pellets formed after culture in media supplemented with TGFβ3 were significantly higher relative to pellets cultured without TGFβ3 supplementation (Figure 1C). The mean GAG content of the pellets cultured under constant hypoxia in the presence of TGFβ3 was 7.8-fold higher (*p* = 0.027) relative to the pellets without TGFβ3 supplementation under hypoxia (Figure 1C). Similarly, under constant normoxia, the pellets cultured with TGFβ3 supplementation were 9.4-fold higher (*p* = 0.001) in mean GAG content relative to those without TGFβ3 (Figure 1C). Moreover, the highest fold change in mean GAG content between pellets cultured with and without TGFβ3 occurred in the hypoxia with reoxygenation experimental group (Figure 1C). The pellets with TGFβ3 supplementation had a 10.2-fold higher (*p* = 0.002) mean GAG content compared with the pellets without TGFβ3 supplementation (Figure 1C).

To determine if cell proliferation may have accounted for the differences between pellets in experimental groups with and without TGFβ3 supplementation, the DNA contents of all pellets were determined (Figure 1D). There was no significant difference in mean DNA contents between pellets cultured in the absence and presence of TGFβ3 under constant hypoxia (Figure 1D). Similarly, there was no significant difference between the mean DNA contents of pellets cultured in the absence and presence of TGFβ3 under hypoxic culture conditions with intermittent reoxygenation periods during *in vitro* culture (Figure 1D). In contrast, the mean DNA content of the pellets cultured with TGFβ3 supplementation under constant normoxia was significantly higher (1.6-fold; *p* = 0.021) than in pellets under normoxia but without TGFβ3 supplementation (Figure 1D).

**FIGURE 2:**
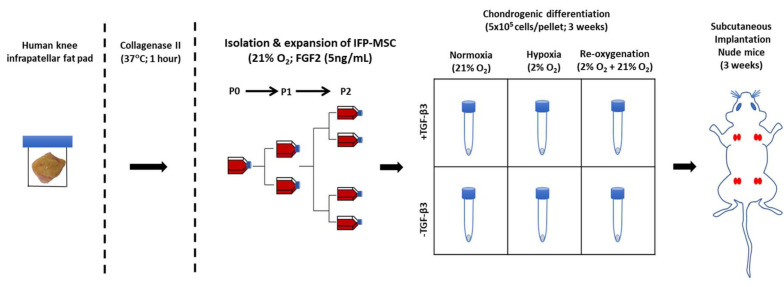
Experimental setup for isolation, expansion, *in vitro* chondrogenesis, and *in vivo* implantation of infrapatellar fat pad-derived MSC (IFP-MSC) in a pellet culture (5 × 10^5^ cells/pellet). Subcutaneous implantation of four groups of cultured pellets in duplicates (same donor) in the back of a male nude mice for 21 days; each group from the same donor was cultured in a different manner than the others (± TGFβ3 supplementation and HYP/NRX/Re-Ox).

As a metric for evaluating the chondrogenic capacity of the pellets formed after *in vitro* chondrogenesis of the IFP-MSC, we determined the GAG/DNA ratio for each pellet (Barbero et al., 2004). There was no significant difference between GAG per DNA contents of the pellets cultured under constant hypoxia regardless of the presence of TGFβ3 (Figure 2). However, there were significant differences between the GAG per DNA content of the pellets cultured with and without TGFβ3 supplementation in the constant normoxia and hypoxia with intermittent reoxygenation experimental groups (Figure 1E). The mean GAG per DNA content of the pellets with TGFβ3 supplementation under constant normoxia was 4.4-fold higher (*p* = 0.012) than in the pellets without TGFβ3. Similarly, the mean GAG per DNA contents of the pellets with TGFβ3 supplementation vs. the GAG per DNA contents of pellets without TGFβ3 supplementation under intermittent reoxygenation culture conditions was significantly 5.4-fold higher (*p* = 0.011) (Figure 1E).

### Induction of Hypoxia-Responsive Genes Is Independent of TGFβ3

**FIGURE 3:**
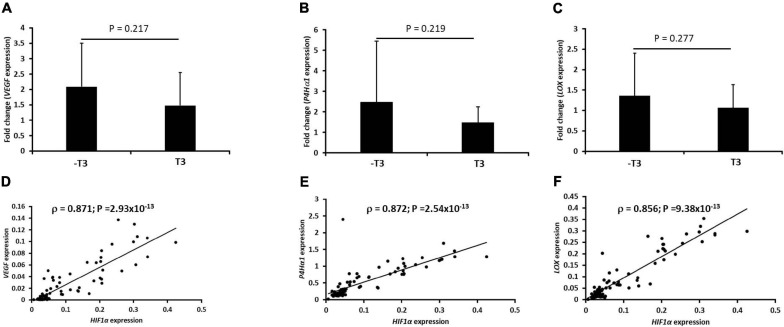
Induction of a biased panel of hypoxia-inducible genes and Spearman correlation plots with hypoxia-inducible factor-1α (*HIF1*α) expression in IFP-MSC pellets. Fold change of mRNA expression of *VEGF*, *P4H*α*1*, and *LOX* for seven donors with independent duplicates (*N* = 7, *n* = 14 per experimental group) under hypoxia (2% O_2_) after normalization to the same gene expression under normoxia (21% O_2_): **(A)** vascular endothelial growth factor (*VEGF*), **(B)** prolyl-4-hydroxylase-α1 (*P4H*α*1*), and **(C)** lysyl oxidase (*LOX*). Spearman correlation of *HIF1*α mRNA with **(D)** *VEGF*, **(E)** *P4H*α*1*, and **(F)** *LOX*. The geometric mean of three housekeeping genes was used for initial normalization: tyrosine 3-monooxygenase/tryptophan 5-monooxygenase activation protein, zeta polypeptide (*YWHAZ*), β-actin (β*-actin*), and β-2-microglobulin (*B2M*) were used. Bar data are mean ± standard deviation. Statistical comparisons were based on unpaired Student’s *t*-test between no TGFβ3 (i.e., –T3) and with TGFβ3 (i.e., +T3) groups.

The expression of *VEGF*, *P4H*α*1*, and *LOX* as hypoxia-responsive genes increased all pellets regardless of whether they were cultured in the presence or absence of chondrogenic media supplemented with TGFβ3 (Figures 3A–C). Furthermore, there was no significant difference in expression of the three genes between the pellets cultured in the presence or absence of TGFβ3. Moreover, the expression of *HIF1*α strongly correlated with the expression of *VEGF*, *P4H*α*1*, and *LOX* (Figures 3D–F). The expression of *HIF1*α strongly correlated with the gene expressions of *VEGF* (Spearman ρ = 0.871; *p*-value = 2.93 × 10^–13^; Figure 3D), *P4H*α*1* (Spearman ρ = 0.872; *p*-value = 2.54 × 10^–13^; Figure 3E), and *LOX* (Spearman ρ = 0.856; *p*-value = 9.38 × 10^–13^; Figure 3E).

### Chondrogenic Gene Induction Is Only Observed With TGFβ3 Supplementation

**FIGURE 4:**
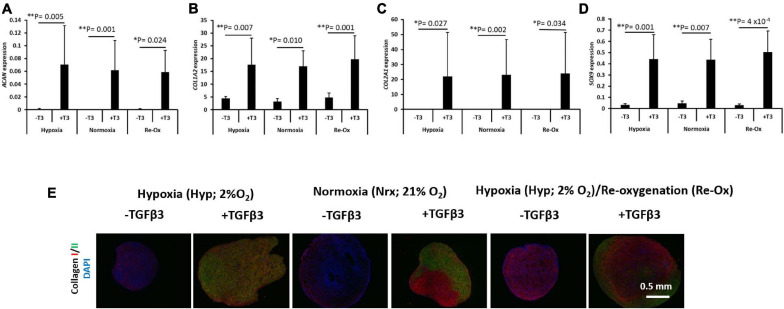
Chondrogenic gene expression and immunofluorescence of types I and II collagen. Relative mRNA expression of **(A)** aggrecan (*ACAN*), **(B)** type I collagen (*COL1A2*), **(C)** type II collagen (*COL2A1*), and **(D)** SRY-Box transcription factor 9 (*SOX9*) for seven donors with independent duplicates (*N* = 7, *n* = 14 per experimental group). Gene expression was normalized to the geometric mean of three housekeeping genes (*YWHAZ*, β*-actin*, and *B2M*). Bar data are mean ± standard deviation. Statistical comparisons were based on Kruskal–Wallis test between without TGFβ3 (i.e., –T3) and with TGFβ3 (i.e., +T3) groups within an oxygen tension group. ^∗^*p*-value < 0.05; ^∗∗^*p*-value < 0.01. **(E)** Immunofluorescence (IF) for collagens I and II and DAPI for cell nuclei. All immunostained pellet sections were prepared at 5 μm.

The expression of a panel of chondrogenic genes including *ACAN*, *COL1A2*, *COL2A1*, and *SOX9* was evaluated in the pellets formed from IFP-MSC after 3 weeks of *in vitro* chondrogenic culture (Figure 4). All pellets formed in the presence of chondrogenic media supplemented with TGFβ3 displayed a significant mRNA expression of *ACAN*, *COL1A2*, *COL2A1*, and *SOX9* regardless of the oxygen tension during *in vitro* culture (Figure 4). Under constant hypoxia, the mean expression of *ACAN* in the pellets cultured with TGFβ3 supplementation was 78-fold higher (*p* = 0.005) than in pellets without TGFβ3 (Figure 4A). Furthermore, under constant normoxia, TGFβ3 induced a significant 429-fold higher mean *ACAN* expression in IFP-MSC pellets relative to pellets without TGFβ3 supplementation (Figure 4A). The mean *ACAN* expression in pellets with TGFβ3 under hypoxia with intermittent reoxygenation was 62-fold higher (*p* = 0.024) relative to pellets under the same oxygen tension but without TGFβ3 supplementation (Figure 4A).

The mean expression of *COL1A2*, *COL2A1*, and *SOX9* similarly followed the same trend as *ACAN* in the pellets with and without TGFβ3 supplementation (Figures 4B–D). *COL1A2* expression was fourfold higher (*p* = 0.007) in pellets with TGFβ3 supplementation relative to pellets without TGFβ3 under constant hypoxia. Under constant normoxia, the mean expression of *COL1A2* was fivefold higher (*p* = 0.001) in pellets with TGFβ3 relative to pellets without TGFβ3 (Figure 4B), while under hypoxia with intermittent reoxygenation, the mean expression of *COL1A2* in pellets with TGFβ3 supplementation was fourfold higher (*p* = 0.010) than in pellets without TGFβ3 supplementation (Figure 4B). The mean expression of *COL2A1* in pellets with TGFβ3 relative to pellets without TGFβ3 supplementation was ∼13,000-fold higher (*p* = 0.027) under constant hypoxia (Figure 4C). Under constant normoxia, the mean expression of *COL2A1* in pellets with TGFβ3 supplementation was 7,413-fold higher (*p* = 0.002) compared with pellets without TGFβ3 (Figure 4C). The mean expression of *COL2A1* in pellets with TGFβ3 treatment was ∼2,875-fold higher (*p* = 0.034) relative to pellets without TGFβ3 treatment under hypoxia with intermittent reoxygenation (Figure 4C). The mean expression of SOX9 in pellets treated with TGFβ3 was 13-fold higher (*p* = 0.001) under constant hypoxia, 9.7-fold higher (*p* = 0.007) under constant normoxia, and ∼17-fold higher (*p* = 4 × 10^–4^) under hypoxia with intermittent reoxygenation, respectively, relative to pellets without TGFβ3 treatment (Figure 4D).

### COLII Immunostaining but Not COLI Is Observed in Pellets With TGFβ3 Supplementation

The pellets were fixed, paraffin wax embedded, and sectioned for immunostaining for types I (COLI) and II (COLII) collagen proteins and DAPI for cell nuclei identification (Figure 4E). COLI immunostaining along with DAPI staining was present in all pellets regardless of the presence of TGFβ3 and the oxygen tension during *in vitro* pellet culture (Figure 4E). In contrast, COLII was observed in pellets that had been cultured in the presence of TGFβ3 supplementation during *in vitro* culture (Figure 4E).

### Hypertrophic Chondrogenesis Gene Induction Was Only Observed With TGFβ3 Supplementation

**FIGURE 5:**
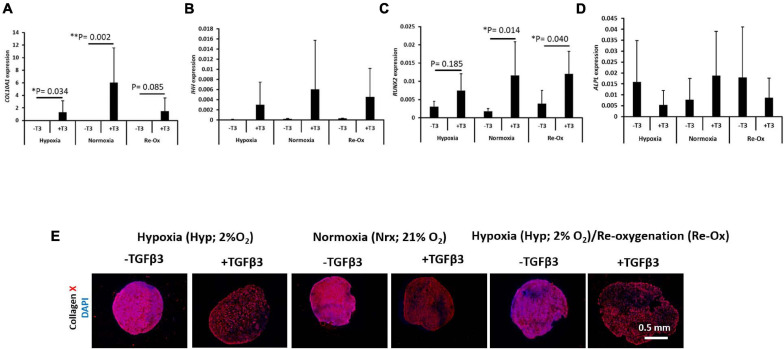
Hypertrophic chondrogenesis marker gene expression and immunofluorescence of type X collagen. Relative mRNA expression of **(A)** type X collagen (*COL10A1*), **(B)** Indian Hedgehog (*IHH*), **(C)** Runt-related transcription factor 2 (*RUNX2*), and **(D)** alkaline phosphatase (*ALPL*) for seven donors with independent duplicates (*N* = 7, *n* = 14 per experimental group). Gene expression was normalized to the geometric mean of three housekeeping genes (*YWHAZ*, β*-actin*, and *B2M*). Bar data are mean ± standard deviation. Statistical comparisons were based on Kruskal–Wallis test between without TGFβ3 (i.e., –T3) and with TGFβ3 (i.e., +T3) groups within an oxygen tension group. ^∗^*p*-value < 0.05; ^∗∗^*p*-value < 0.01. **(E)** Immunofluorescence (IF) for type X collagen and DAPI for cell nuclei. All immunostained pellet sections were prepared at 5 μm.

The expression of a panel of hypertrophic chondrogenesis-related genes including *COL10A1*, *IHH*, *RUNX2*, and *ALPL* was evaluated in IFP-MSC pellets after 3 weeks of *in vitro* chondrogenesis (Figure 5). The mean expression of *COL10A1* was 5,550-fold higher (*p* = 0.034) in pellets treated with TGFβ3 relative to those without TGFβ3 treatment under constant hypoxia (Figure 5). Similarly, the mean expression of *COL10A1* was 5,155-fold higher (*p* = 0.002) in pellets with TGFβ3 supplementation relative to those without supplementation under constant normoxia (Figure 5A). However, there was no statistical difference between the mean expression of *COL10A1* in pellets with and without TGFβ3 treatment under hypoxia with intermittent reoxygenation during *in vitro* culture (Figure 5A). The mean expression of *IHH* and *ALPL* was not significantly different between pellets with and without TGFβ3 supplementation within the same oxygen tension and across all experimental groups (Figures 5B,D). The mean expression of *RUNX2* in pellets with TGFβ3 supplementation under constant hypoxia was not significantly different from those without TGFβ3 supplementation (Figure 5C). However, the mean expression of *RUNX2* in the pellets treated with TGFβ3 under constant normoxia was significantly 6.4-fold higher (*p* = 0.014) than in the pellets without TGFβ3 supplementation (Figure 5C). Similarly, the mean expression of *RUNX2* was significantly 3.1-fold higher in pellets treated with TGFβ3 relative to those without TGFβ3 treatment under hypoxia with intermittent reoxygenation (Figure 5C).

### COLX Immunostaining Is Observed in Pellets With and Without TGFβ3 Supplementation

The pellets were fixed, paraffin wax embedded, and sectioned as before but tested immunofluorescently for type X (COLX) collagen and DAPI for cell nuclei identification (Figure 5E). COLX immunostaining was present in all pellets regardless of TGFβ3 supplementation and the oxygen tension during *in vitro* culture (Figure 5E). The COLX staining was diffusely distributed across the pellets formed with TGFβ3 supplementation during *in vitro* culture. In contrast, the COLX staining in the pellets without TGFβ3 supplementation appeared to be densely distributed (Figure 5E).

### *TGF*β1 and *TGF*β3 Genes Are Induced but Not TGFβ2 in Pellets With TGFβ3 Supplementation

**FIGURE 6:**
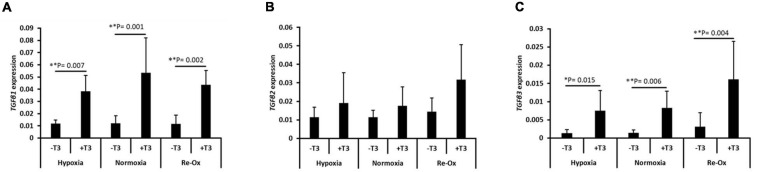
Induction of the gene expression of transforming growth factor-β (TGFβ) isoforms in IFP-MSC pellets after 21 days of *in vitro* culture. Relative mRNA expression of **(A)** *TGFβ1*, **(B)** *TGFβ2*, and **(C)** *TGFβ3* for seven donors with independent duplicates (*N* = 7, *n* = 14 per experimental group). Gene expression was normalized to the geometric mean of three housekeeping genes (*YWHAZ*, β*-actin*, and *B2M*). Bar data are mean ± standard deviation. Statistical comparisons were based on Kruskal–Wallis test between without TGFβ3 (i.e., –T3) and with TGFβ3 (i.e., +T3) supplementation groups within an oxygen tension group. ^∗^*p*-value < 0.05; ^∗∗^*p*-value < 0.01.

Hypoxia at 2% O_2_ induced *in vitro* chondrogenesis of bone marrow-derived mesenchymal stem cells (BM-MSC) in the absence of exogenous TGFβ3. The induction was associated with significant upregulation of *TGFβ1* expression (Marsano et al., 2016). We therefore investigated the gene expression of the three isoforms of TGFβ in pellets across the different experimental groups (Figure 6). The mean expression of *TGFβ1* and *TGFβ3* was significantly higher in pellets formed with TGFβ3 supplementation during culture relative to pellets without exogenous TGFβ3 regardless of the oxygen tension (Figures 6A,C). However, *TGFβ2* expression was not statistically different across experimental groups (Figure 6B). The mean expression of *TGFβ1* was significantly higher (3.3-fold; *p* = 0.007) in pellets with TGFβ3 under constant hypoxia relative to those without TGFβ3 supplementation (Figure 6A). Similarly, under constant normoxia, the mean expression of *TGFβ1* was significantly higher (4.4-fold; *p* = 0.001) in pellets with TGFβ3 supplementation compared with pellets without the supplementation (Figure 6A). A similar trend also continued in pellets cultured under hypoxia with intermittent reoxygenation. The mean expression of *TGFβ1* in pellets with TGFβ3 supplementation was 3.8-fold higher (*p* = 0.002) relative to the pellets without TGFβ3 supplementation (Figure 6A). The mean expression of *TGFβ3* was 5.4-fold higher (*p* = 0.015) under constant hypoxia, 5.9-fold higher (*p* = 0.006) under constant normoxia, and 5.2-fold higher (*p* = 0.004) under hypoxia with intermittent reoxygenation in pellets supplemented with exogenous TGFβ3 during culture relative to pellets under the respective oxygen conditions but without TGFβ3 supplementation (Figure 6C).

### Normoxia-Cultured Pellets in the Presence of TGFβ3 Retained Chondrogenic Phenotype *in vivo*

**FIGURE 7:**
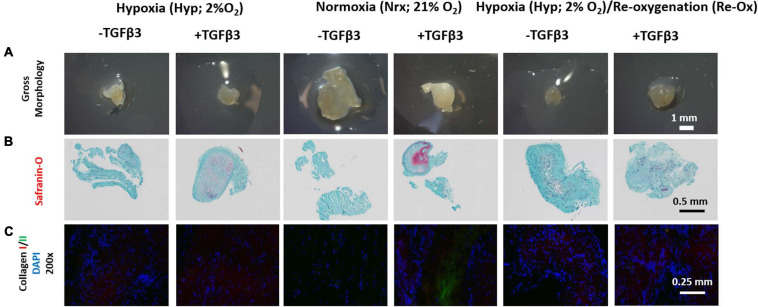
Gross morphology of IFP-MSC pellets after 3 weeks of *in vivo* implantation in nude mice; Safranin O histochemical staining and immunofluorescence (IF) for types I and II collagen. **(A)** Gross morphology of pellets; **(B)** Safranin O staining; **(C)** IF for collagen I, II, and DAPI, for cell nuclei (×200 magnification). All Safranin O and immunostained pellet sections were prepared at 5 μm.

The pellets from the different pretreatment groups differed in gross appearance after 3 weeks of subcutaneous implantation in mice (Figure 7A). Except for the pellets that were precultured under normoxia in the presence of TGFβ3 which appeared opaque, the remaining pellets appeared somewhat translucent (Figure 7A). The pellets that were precultured in the presence of TGFβ3 under hypoxia and normoxia were positive for Safranin O but not the pellets that underwent intermittent reoxygenation (Figure 7B). Moreover, except for the pellets that were precultured under normoxia in the presence of TGFβ3, there was trace to no COLII immunofluorescence in the rest of the pellets (Figure 7C and Supplementary Figure 1). All pellets were positive for COLI immunofluorescence albeit with varied relative fluorescence unit (Figure 7C and Supplementary Figure 1).

### Collagen X Is Present but No Calcification in All *in vivo* Implanted Pellets

**FIGURE 8:**
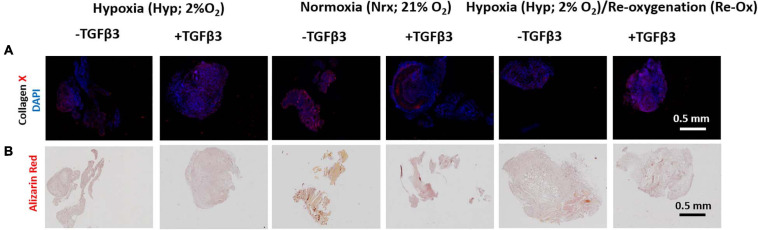
Immunofluorescence (IF) and Alizarin Red S histochemical analysis of IFP-MSC pellets after 3 weeks of *in vivo* implantation in nude mice. **(A)** IF for collagen X and DAPI for cell nuclei; **(B)** Alizarin Red S staining for calcification. All immunostained and Alizarin Red S pellet sections were prepared at 5 μm.

All pellets retained type X collagen (COLX) deposits after 3 weeks of implantation in mice but with varied levels between experimental groups (Figure 8A and Supplementary Figure 2). There was little to no evidence of calcification in the pellets based on the lack of Alizarin Red S staining after 3 weeks of subcutaneous implantation in mice (Figure 8B).

### Principal Component Analysis Shows a Predominantly Hypertrophic Chondrogenesis

**FIGURE 9:**
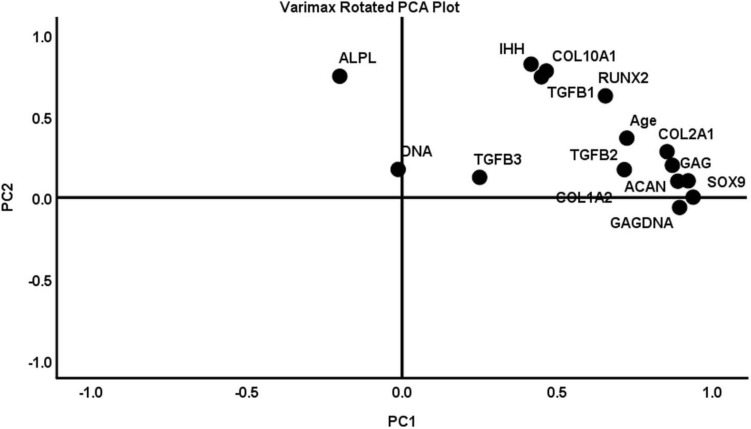
A varimax rotated plot of a two-component solution of principal component analysis of measured variables. Measured variables are as follows: *ACAN*, age, *ALPL*, *COL1A2*, *COL2A1*, *COL10A1*, DNA, GAG, GAG/DNA (GAGDNA), *IHH*, *RUNX2*, *SOX9*, *TGFβ1*, *TGFβ2*, and *TGFβ3*. Principal component 1 (PC1) is on the *x*-axis and principal component 2 (PC2) is on the *y*-axis. The position of each variable on the plot indicates the degree to which it loads on the two different principal components.

Principal component analysis (PCA) was used to summarize pooled biochemical and gene expression data and determine the relationships between the measured variables (Figure 9). Initial PCA generated a four-component solution with eigenvalues exceeding 1: PC1, PC2, PC3, and PC4. The Kaiser–Meyer–Olkin (KMO) measure of sampling adequacy to implement a PCA was 0.570. However, two of the components (i.e., PC3 and PC4) accounted for less than 10% of the variance in the pooled data, and the remaining two—PC1 and PC2—accounted for 50 and 13%, respectively. Therefore, the PCA was performed again to extract a two-component solution of PC1 and PC2. The KMO measure of sampling adequacy remained unchanged and CPD was equally aligned to PC1 and PC2. Therefore, CPD was removed as a variable and the PCA was ran again for a two-component solution. The KMO was 0.689 (Table 2). PC1 and PC2 accounted for 53 and ∼14% of the variance in the pooled data, respectively. PC1 was largely characterized by metrics of chondrogenesis: GAG, GAG/DNA, *ACAN*, *COL1A2*, *COL2A1*, and *SOX9*. In contrast, PC2 was characterized by variables associated with hypertrophic chondrogenesis: *IHH*, *COL10A1*, and *ALPL* (Table 3). Moreover, *TGFβ1* correlated with PC2, while *TGFβ2* and *TGFβ3* aligned more closely with PC1 than PC2 (Table 3).

**TABLE 2:** Kaiser–Meyer–Olkin and Bartlett’s test for principal component analysis (PCA).

KMO and Bartlett’s test		
Kaiser–Meyer–Olkin measure of sampling adequacy		0.689
Bartlett’s test of sphericity	Approx. chi-square	432.300
	*df*	105
	Sig.	2.73E–41

**TABLE 3:** Principal component analysis (PCA) correlation matrix.

	PC1	PC2
Age	0.724	0.365
GAG	0.887	0.100
DNA	–0.013	0.173
GAGDNA	0.893	–0.061
*ACAN*	0.870	0.198
*COL1A2*	0.937	0.002
*COL2A1*	0.853	0.282
*COL10A1*	0.464	0.778
*SOX9*	0.921	0.102
*TGFB1*	0.448	0.742
*TGFB3*	0.250	0.124
*TGFB2*	0.715	0.171
*IHH*	0.415	0.820
*RUNX2*	0.655	0.625
*ALPL*	–0.200	0.746

## Discussion

The IFP represents an arthroscopically accessible source of MSC for the repair of articular cartilage defects due to the high proliferative and chondrogenic potential of IFP-MSC within it (Wickham et al., 2003; Khan et al., 2007). However, *in vitro* chondrogenic protocols for IFP-MSC are either a direct transfer or a variant of protocols developed for BM-MSC and involve exogenous additions of TGFβ (Johnstone et al., 1998; Wickham et al., 2003; Khan et al., 2007; Buckley et al., 2011; Buckley and Kelly, 2012). In this study, we explored a low oxygen tension (hypoxia) of 2% as a driver of chondrogenic differentiation of IFP-MSC. We had hypothesized that 2% O_2_ would induce chondrogenesis in the IFP-MSC pellets based on the findings of Marsano et al. (2016) with FGF2-amplified human BM-MSC, which demonstrated that hypoxia at 2% O_2_ alone was sufficient to induce the *in vitro* chondrogenesis of the BM-MSC. We embarked to test the hypothesis using the pellet model of *in vitro* chondrogenesis MSC both in the presence and absence of TGFβ3 (Johnstone et al., 1998; Marsano et al., 2016).

Firstly, we demonstrated and validated the reality of hypoxia signaling in our pellet model under 2% O_2_ atmosphere. Our results showed that the IFP-MSC experienced a significant induction of hypoxia-responsive gene expression (HRE) of *VEGF*, *PH4*α*1*, and *LOX* as proof that the IFP-MSC experienced hypoxia relative to control IFP-MSC at 21% O_2_ (Semenza, 2001; Myllyharju, 2008; van Vlimmeren et al., 2010; Makris et al., 2014). Moreover, the expression of *HIF1*α correlated positively with the expression of the HREs, suggesting that the transcriptional activity of HIF1 was involved in the hypoxic response of the FGF2-amplified human IFP-MSC (Semenza, 2001).

We confirmed *in vitro* chondrogenesis or the lack of it through gene expression of chondrogenic markers, biochemical (i.e., GAG/DNA) and histological assessments of cartilage-specific matrix (i.e., Safranin O, COLI, II, and X) in the microtissue pellets of the human IFP-MSC. Overall, our findings demonstrated that 2% O_2_ alone was inadequate to induce chondrogenesis in FGF2-expanded human IFP-MSC. The reason for this may be primarily due to the lack of induction of TGFβ1 or TGFβ3 by the 2% O_2_, relative to the pellets of the same FGF2-expanded human IFP-MSC which underwent chondrogenesis in the presence of TGFβ3 supplementation (Figures 6A,C). Mechanistically, Marsano et al. (2016) had demonstrated that pure 2% O_2_ induced *TGFβ1* expression to affect the *in vitro* chondrogenesis of FGF2-expanded human BM-MSC without TGFβ3 supplementation. In this study, we observed significant induction of *TGFβ1* and *TGFβ3* mRNA along with evidence of chondrogenesis in the pellets of FGF2-expanded human IPF-MSC but only in the presence of TGFβ3 supplementation.

Thus, our findings suggest that the drivers of the *in vitro* chondrogenesis of the FGF2-expanded human IFP-MSC involved both *TGFβ1*- and *TGFβ3*-mediated signaling. Moreover, PCA revealed that *TGFβ1* and *TGFβ3* induction correlated more strongly with PC2 and PC1, respectively. Given that PC2 is characterized by the key markers/metrics of unstable hypertrophic chondrogenesis (*IHH*, *COL10A1*, and *ALPL*) (van der Kraan and van den Berg, 2012), while PC1 correlated more strongly with the markers of stable chondrogenesis (*SOX9*, *ACAN*, *COL2A1*, GAG, and GAG/DNA), it opens the perspective that selective inhibition of TGFβ1 during the *in vitro* chondrogenesis of FGF2-expanded human IFP-MSC under 2% O_2_ may result in stable chondrogenesis rather than to unstable hypertrophic chondrogenesis. However, the selective TGFβ1 inhibitor must outperform the local and high affinity interactions between TGFβ1 and its heteromeric TGFβ receptor complex (Bedinger et al., 2016; Martin et al., 2020).

The premature induction of markers of chondrocyte hypertrophy-related molecules such as type X collagen (*COL10A1*) and matrix metalloproteinase 13 (*MMP13*) prior to type II collagen (*COL2A1*) in human BM-MSC undergoing *in vitro* chondrogenesis in the presence of TGFβ3 correlated with the transformation of the chondrogenically induced human BM-MSC to bone-like tissue after 4–6 weeks of subcutaneous implantation in immunodeficient mice (Pelttari et al., 2006). While this study did not include a time course evaluation of the expression of type II collagen and type X collagen prior to the subcutaneous implantation of the chondrogenically induced IFP-MSC in immunodeficient mice, it revealed some mixed findings of Pelttari et al. (2006).

Our data supported the retention of Safranin O-positive proteoglycan and type II collagen matrix after *in vivo* implantation but only in the context of pellets formed under 21% O_2_ with TGFβ3 supplementation (Figures 7B,C), as reported also by Pelttari et al. (2006). There was retention of Safranin O-positive proteoglycan matrix after *in vivo* implantation of the pellets formed under hypoxia in the presence of TGFβ3 (Figures 7B,C), but this lacked the initial deposits of type II collagen prior to the implantation (Figure 4E). The mechanism underlying these findings is unclear but seem to suggest that the oxygen tension during the *in vitro* chondrogenesis of IFP-MSC may impact the stability of engineered cartilage *in vivo*. One possibility may be related to hypoxia-induced expression of the chemotactic agent, stromal derived factor-1 (SDF-1), which has been shown to facilitate the recruitment of inflammatory cells such as monocyte/macrophages, leading to subsequent breakdown of cartilage matrix (Hitchon et al., 2002; Ceradini et al., 2004; Schantz et al., 2007; Elkington et al., 2009; Lau and Wang, 2011; Sánchez-Martín et al., 2011). However, the existence of SDF-1 and its potential role in the loss of the type II collagen in the ECM of the IFP-MSC pellets formed under 2% O_2_ with TGFβ3 supplementation need to be verified.

In contrast to the findings of Pelttari et al. (2006), there was no evidence of calcification of the pellets in this study after subcutaneous implantation regardless of whether the IFP-MSC pellets were chondrogenically stimulated under 21% O_2_ or 2% O_2_ in the presence of TGFβ3 supplementation. This finding was unexpected and even surprising given the presence of type X collagen in the pellets. Type X collagen is known to bind calcium and participate in the calcification of growth plate cartilage during endochondral ossification (Kirsch and von der Mark, 1991). However, it is worthy of mention that the induction and deposition of type X collagen in the IFP-MSC pellets lacking chondrogenic induction was reported in the cell culture-amplified human BM-MSC (Barry et al., 2001; Tuli et al., 2003). This suggests that the mechanisms underlying type X collagen expression in MSC from bone marrow and adipose tissue are perhaps similar and independent of chondrogenic differentiation. Therefore, the association of type X collagen expression in *in vitro* cultured MSC with ectopic calcification may be more complex than previously thought. Moreover, there is the possibility that the 3 weeks of ectopic implantation of the pellets in this study vs. the 4–6 weeks of implantation by Pelttari et al. (2006) may have been a factor in the lack of observed calcification. It is also noteworthy that the human BM-MSC in the study of Pelttari et al. (2006) were sourced from patients at late stage of hip osteoarthritis, which Mwale et al. (2006) also demonstrated to express type X collagen prior to chondrogenic stimulation.

In conclusion, low oxygen tension of 2% O_2_ alone was unable to induce chondrogenesis in micromass pellet cultures of IFP-MSC. This finding contrasts with the findings of Marsano et al. (2016) which demonstrated that the same oxygen tension was capable of inducing chondrogenesis in pellet cultures of human BM-MSC. Moreover, the cartilage microtissues formed under normoxia with TGFβ3 supplementation retained their chondrogenic phenotype *in vivo* relative to their hypoxia counterparts. However, given the existence of two distinct populations of IFP-MSC within the IFP and their different chondrogenic potential (Hindle et al., 2017), further investigation is merited on the chondroinductive potential of low oxygen tension on the distinct populations of IFP-MSC.

## Data Availability Statement

The raw data supporting the conclusions of this article will be made available by the authors, without undue reservation.

## Ethics Statement

The studies involving human tissue specimens were reviewed and approved by the University of Alberta’s Health Research Ethics Board-Biomedical Panel. Written informed consent for participation was not required for this study in accordance with the national legislation and the institutional requirements. The animal study was reviewed and approved by the University of Alberta Animal Care and Use Committee.

## Author Contributions

SR conducted the bulk of the experiments and was responsible for data acquisition, analysis, and initial manuscript writing. AS assisted with the experiments conducted in the hypoxia workstation (i.e., X3 Xvivo system, BioSpherix). YL performed the animal surgery with assistance from MK. MK was responsible for performing qPCR and gene expression analysis. AM-S assisted with the experiment setup and immunofluorescence assays. VG performed DNA and histological assays. NJ was responsible for IFP procurement and manuscript review. AA conceived and supervised the study, performed PCA and other statistical analysis, and was responsible for the writing and the final review of the manuscript. All authors read and approved the final manuscript.

## Conflict of Interest

The authors declare that the research was conducted in the absence of any commercial or financial relationships that could be construed as a potential conflict of interest.

## Publisher’s Note

All claims expressed in this article are solely those of the authors and do not necessarily represent those of their affiliated organizations, or those of the publisher, the editors and the reviewers. Any product that may be evaluated in this article, or claim that may be made by its manufacturer, is not guaranteed or endorsed by the publisher.

## References

[B1] Afizah H. Yang Z. Hui J. H. Ouyang H. W. Lee E. H. (2007). A comparison between the chondrogenic potential of human bone marrow stem cells (BMSCs) and adipose-derived stem cells (ADSCs) taken from the same donors. *Tissue Eng.* 13 659–666. 10.1089/ten.2006.0118 17371203

[B2] Awad H. A. Halvorsen Y. D. Gimble J. M. Guilak F. (2003). Effects of transforming growth factor beta1 and dexamethasone on the growth and chondrogenic differentiation of adipose-derived stromal cells. *Tissue Eng.* 9 1301–1312. 10.1089/10763270360728215 14670117

[B3] Barbero A. Grogan S. Schafer D. Heberer M. Mainil-Varlet P. Martin I. (2004). Age related changes in human articular chondrocyte yield, proliferation and post-expansion chondrogenic capacity. *Osteoarthritis Cartilage* 12 476–484. 10.1016/j.joca.2004.02.010 15135144

[B4] Barry F. Boynton R. Liu B. Murphy J. (2001). Chondrogenic differentiation of mesenchymal stem cells from bone marrow: differentiation-dependent gene expression of matrix components. *Exp. Cell Res.* 268 189–200. 10.1006/excr.2001.5278 11478845

[B5] Bedinger D. Lao L. Khan S. Lee S. Takeuchi T. Mirza A. M. (2016). Development and characterization of human monoclonal antibodies that neutralize multiple TGFbeta isoforms. *MABS* 8 389–404. 10.1080/19420862.2015.1115166 26563652 PMC4966579

[B6] Bornes T. D. Adesida A. B. Jomha N. M. (2014). Mesenchymal stem cells in the treatment of traumatic articular cartilage defects: a comprehensive review. *Arthritis Res.Ther.* 16:432. 10.1186/s13075-014-0432-1 PMC4289291 25606595

[B7] Bornes T. D. Adesida A. B. Jomha N. M. (2018). Articular Cartilage Repair with Mesenchymal Stem Cells After Chondrogenic Priming: a Pilot Study. *Tissue Eng. Part A* 24 761–774. 10.1089/ten.tea.2017.0235 28982297

[B8] Brighton C. T. Heppenstall R. B. (1971b). Oxygen tension in zones of the epiphyseal plate, the metaphysis and diaphysis. An in vitro and in vivo study in rats and rabbits. *J. Bone Joint Surg. Am.* 53 719–728. 10.2106/00004623-197153040-00011 5580029

[B9] Brighton C. T. Heppenstall R. B. (1971a). Oxygen tension of the epiphyseal plate distal to an arteriovenous fistula. *Clin. Orthop. Relat. Res.* 80 167–173. 10.1097/00003086-197110000-00024 5133323

[B10] Brittberg M. Lindahl A. Nilsson A. Ohlsson C. Isaksson O. Peterson L. (1994). Treatment of deep cartilage defects in the knee with autologous chondrocyte transplantation. *N. Engl. J. Med.* 331 889–895. 8078550 10.1056/NEJM199410063311401

[B11] Brittberg M. Tallheden T. Sjorgren-Jansson E. Lindahl A. Peterson L. (2001). Autologous chondrocytes used for articular cartilage repair. *Clin. Orthop. Relat. Res.* 391 S337–S348. 10.1097/00003086-200110001-00031 11603717

[B12] Buckley C. T. Kelly D. J. (2012). Expansion in the presence of FGF-2 enhances the functional development of cartilaginous tissues engineered using infrapatellar fat pad derived MSCs. *J. Mech. Behav. Biomed. Mater.* 11 102–111. 10.1016/j.jmbbm.2011.09.004 22658159

[B13] Buckley C. T. Vinardell T. Kelly D. J. (2011). Oxygen tension differentially regulates the functional properties of cartilaginous tissues engineered from infrapatellar fat pad derived MSCs and articular chondrocytes. *Osteoarthritis Cartilage* 18 1345–1354. 10.1016/j.joca.2010.07.004 20650328

[B14] Ceradini D. J. Kulkarni A. R. Callaghan M. J. Tepper O. M. Bastidas N. Kleinman M. E. (2004). Progenitor cell trafficking is regulated by hypoxic gradients through HIF-1 induction of SDF-1. *Nat. Med.* 10 858–864. 10.1038/nm1075 15235597

[B15] Cheng N. C. Estes B. T. Awad H. A. Guilak F. (2009). Chondrogenic differentiation of adipose-derived adult stem cells by a porous scaffold derived from native articular cartilage extracellular matrix. *Tissue Eng. Part A* 15 231–241. 10.1089/ten.tea.2008.0253 18950290 PMC3592270

[B16] Choi J. R. Pingguan-Murphy B. Wan Abas W. A. Noor Azmi M. A. Omar S. Z. Chua K. H. (2014). Impact of low oxygen tension on stemness, proliferation and differentiation potential of human adipose-derived stem cells. *Biochem. Biophys. Res. Commun.* 448 218–224. 10.1016/j.bbrc.2014.04.096 24785372

[B17] Diekman B. O. Rowland C. R. Lennon D. P. Caplan A. I. Guilak F. (2010). Chondrogenesis of adult stem cells from adipose tissue and bone marrow: induction by growth factors and cartilage-derived matrix. *Tissue Eng. Part A* 16 523–533. 10.1089/ten.tea.2009.0398 19715387 PMC2813149

[B18] Ding D. C. Wu K. C. Chou H. L. Hung W. T. Liu H. W. Chu T. Y. (2015). Human Infrapatellar Fat Pad-Derived Stromal Cells Have More Potent Differentiation Capacity Than Other Mesenchymal Cells and Can Be Enhanced by Hyaluronan. *Cell Transplant.* 24 1221–1232. 10.3727/096368914x681937 24853696

[B19] Elkington P. T. Green J. A. Friedland J. S. (2009). “Analysis of Matrix Metalloproteinase Secretion by Macrophages”. *In Macrophages and Dendritic Cells. Methods in Molecular Biology^TM^ (Methods and Protocols)*, eds Reiner N. (Totowa: Humana Press). 10.1007/978-1-59745-396-7_16 19347322

[B20] Erickson G. R. Gimble J. M. Franklin D. M. Rice H. E. Awad H. Guilak F. (2002). Chondrogenic potential of adipose tissue-derived stromal cells in vitro and in vivo. *Biochem. Biophys. Res. Commun.* 290 763–769. 10.1006/bbrc.2001.6270 11785965

[B21] Estes B. Wu A. Guilak F. (2006). Potent induction of chondrocytic differentiation of human adipose-derived adult stem cells by bone morphogenetic protein 6. *Arthritis Rheum.* 54 1222–1232. 10.1002/art.21779 16572454

[B22] Farndale R. W. Buttle D. J. Barrett A. J. (1986). Improved quantitation and discrimination of sulphated glycosaminoglycans by use of dimethylmethylene blue. *Biochim. Biophys. Acta* 883 173–177. 10.1016/0304-4165(86)90306-5 3091074

[B23] Fellows C. R. Matta C. Zakany R. Khan I. M. Mobasheri A. (2016). Adipose, Bone Marrow and Synovial Joint-Derived Mesenchymal Stem Cells for Cartilage Repair. *Front. Genet.* 7:213. 10.3389/fgene.2016.00213 28066501 PMC5167763

[B24] Foldager C. B. Munir S. Ulrik-Vinther M. Soballe K. Bunger C. Lind M. (2009). Validation of suitable house keeping genes for hypoxia-cultured human chondrocytes. *BMC Mol. Biol.* 10:94. 10.1186/1471-2199-10-94 19818117 PMC2764705

[B25] Freitag J. Shah K. Wickham J. Li D. Norsworthy C. Tenen A. (2020). Evaluation of autologous adipose-derived mesenchymal stem cell therapy in focal chondral defects of the knee: a pilot case series. *Regen. Med.* 15 1703–1717. 10.2217/rme-2020-0027 32735154

[B26] Hennig T. Lorenz H. Thiel A. Goetzke K. Dickhut A. Geiger F. (2007). Reduced chondrogenic potential of adipose tissue derived stromal cells correlates with an altered TGFbeta receptor and BMP profile and is overcome by BMP-6. *J. Cell Physiol.* 211 682–691. 10.1002/jcp.20977 17238135

[B27] Hindle P. Khan N. Biant L. Péault B. (2017). The Infrapatellar Fat Pad as a Source of Perivascular Stem Cells with Increased Chondrogenic Potential for Regenerative Medicine. *Stem Cells Transl. Med.* 6 77–87. 10.5966/sctm.2016-0040 28170170 PMC5442731

[B28] Hitchon C. Wong K. Ma G. Reed J. Lyttle D. El-Gabalawy H. (2002). Hypoxia-induced production of stromal cell-derived factor 1 (CXCL12) and vascular endothelial growth factor by synovial fibroblasts. *Arthritis Rheum.* 46 2587–2597. 10.1002/art.10520 12384916

[B29] Huang J. I. Zuk P. A. Jones N. F. Zhu M. Lorenz H. P. Hedrick M. H. (2004). Chondrogenic potential of multipotential cells from human adipose tissue. *Plast. Reconstr. Surg.* 113 585–594. 10.1097/01.prs.0000101063.27008.e1 14758221

[B30] Hwang N. S. Im S. G. Wu P. B. Bichara D. A. Zhao X. Randolph M. A. (2011). Chondrogenic Priming Adipose-Mesenchymal Stem Cells for Cartilage Tissue Regeneration. *Pharm. Res.* 28 1395–1405. 10.1007/s11095-011-0445-2 21494923

[B31] Johnstone B. Hering T. M. Caplan A. I. Goldberg V. M. Yoo J. U. (1998). In vitro chondrogenesis of bone marrow-derived mesenchymal progenitor cells. *Exp. Cell Res.* 238 265–272. 9457080 10.1006/excr.1997.3858

[B32] Khan W. S. Adesida A. B. Hardingham T. E. (2007). Hypoxic conditions increase hypoxia-inducible transcription factor 2alpha and enhance chondrogenesis in stem cells from the infrapatellar fat pad of osteoarthritis patients. *Arthritis Res. Ther.* 9:R55. 10.1186/ar2211 PMC2206341 17537234

[B33] Kim H. J. Im G. I. (2009). Chondrogenic differentiation of adipose tissue-derived mesenchymal stem cells: greater doses of growth factor are necessary. *J. Orthop. Res.* 27 612–619. 10.1002/jor.20766 18985688

[B34] Kirsch T. von der Mark K. (1991). Ca2+ binding properties of type X collagen. *FEBS Lett.* 294 149–152. 10.1016/0014-5793(91)81363-d 1743285

[B35] Lau T. T. Wang D. A. (2011). Stromal cell-derived factor-1 (SDF-1): homing factor for engineered regenerative medicine. *Expert Opin. Biol. Ther.* 11 189–197. 10.1517/14712598.2011.546338 21219236

[B36] Lund-Olesen K. (1970). Oxygen tension in synovial fluids. *Arthritis Rheum.* 13 769–776. 10.1002/art.1780130606 5495389

[B37] Makris E. A. Responte D. J. Paschos N. K. Hu J. C. Athanasiou K. A. (2014). Developing functional musculoskeletal tissues through hypoxia and lysyl oxidase-induced collagen cross-linking. *Proc. Natl. Acad. Sci. U. S. A.* 111 E4832–E4841. 25349395 10.1073/pnas.1414271111 PMC4234579

[B38] Malladi P. Xu Y. Chiou M. Giaccia A. J. Longaker M. T. (2007). Hypoxia inducible factor-1alpha deficiency affects chondrogenesis of adipose-derived adult stromal cells. *Tissue Eng.* 13 1159–1171. 10.1089/ten.2006.0265 17518738

[B39] Marsano A. Medeiros Da Cunha C. M. Ghanaati S. Gueven S. Centola M. Tsaryk R. (2016). Spontaneous In Vivo Chondrogenesis of Bone Marrow-Derived Mesenchymal Progenitor Cells by Blocking Vascular Endothelial Growth Factor Signaling. *Stem Cells Transl. Med.* 5 1730–1738. 10.5966/sctm.2015-0321 27460852 PMC5189644

[B40] Martin C. J. Datta A. Littlefield C. Kalra A. Chapron C. Wawersik S. (2020). Selective inhibition of TGFβ1 activation overcomes primary resistance to checkpoint blockade therapy by altering tumor immune landscape. *Sci. Transl. Med.* 12:eaay8456. 10.1126/scitranslmed.aay8456 32213632

[B41] Martin J. A. Buckwalter J. A. (2003). The role of chondrocyte senescence in the pathogenesis of osteoarthritis and in limiting cartilage repair. *J. Bone Joint Surg. Am.* 85-A 106–110. 10.2106/00004623-200300002-00014 12721352

[B42] Martinez-Lorenzo M. J. Royo-Canas M. Alegre-Aguaron E. Desportes P. Castiella T. Garcia-Alvarez F. (2009). Phenotype and chondrogenic differentiation of mesenchymal cells from adipose tissue of different species. *J. Orthop. Res.* 27 1499–1507. 10.1002/jor.20898 19408284

[B43] Mehlhorn A. T. Niemeyer P. Kaschte K. Muller L. Finkenzeller G. Hartl D. (2007). Differential effects of BMP-2 and TGF-beta1 on chondrogenic differentiation of adipose derived stem cells. *Cell Prolif.* 40 809–823. 10.1111/j.1365-2184.2007.00473.x 18021172 PMC6496220

[B44] Merceron C. Vinatier C. Portron S. Masson M. Amiaud J. R. M. Guigand L. (2010). Differential effects of hypoxia on osteochondrogenic potential of human adipose-derived stem cells. *Am. J. Physiol. Cell Physiol.* 298 C355–C364. 19940068 10.1152/ajpcell.00398.2009

[B45] Mobasheri A. Kalamegam G. Musumeci G. Batt M. E. (2014). Chondrocyte and mesenchymal stem cell-based therapies for cartilage repair in osteoarthritis and related orthopaedic conditions. *Maturitas* 78 188–198. 10.1016/j.maturitas.2014.04.017 24855933

[B46] Munir S. Foldager C. Lind M. Zachar V. Søballe K. Koch T. (2014). Hypoxia enhances chondrogenic differentiation of human adipose tissue-derived stromal cells in scaffold-free and scaffold systems. *Cell Tissue Res.* 355 89–102. 10.1007/s00441-013-1732-5 24178804

[B47] Muthuri S. G. Mcwilliams D. F. Doherty M. Zhang W. (2011). History of knee injuries and knee osteoarthritis: a meta-analysis of observational studies. *Osteoarthritis Cartilage* 19 1286–1293. 10.1016/j.joca.2011.07.015 21884811

[B48] Mwale F. Stachura D. Roughley P. Antoniou J. (2006). Limitations of using aggrecan and type X collagen as markers of chondrogenesis in mesenchymal stem cell differentiation. *J. Orthop. Res.* 24 1791–1798. 10.1002/jor.20200 16779832

[B49] Myllyharju J. (2008). Prolyl 4-hydroxylases, key enzymes in the synthesis of collagens and regulation of the response to hypoxia, and their roles as treatment targets. *Ann. Med.* 40 402–417. 10.1080/07853890801986594 19160570

[B50] Pelttari K. Winter A. Steck E. Goetzke K. Hennig T. Ochs B. G. (2006). Premature induction of hypertrophy during in vitro chondrogenesis of human mesenchymal stem cells correlates with calcification and vascular invasion after ectopic transplantation in SCID mice. *Arthritis Rheum.* 54 3254–3266. 10.1002/art.22136 17009260

[B51] Peterson L. Minas T. Brittberg M. Nilsson A. Sjorgren-Jansson E. Lindahl A. (2000). two- to 9- year outcome after autologous chondrocyte transplantation of the knee. *Clin. Orthop. Relat. Res.* 374 212–234. 10.1097/00003086-200005000-00020 10818982

[B52] Provot S. Schipani E. (2007). Fetal Growth Plate: a Developmental Model of Cellular Adaptation to Hypoxia. *Ann. N. Y. Acad. Sci.* 1117 26–39. 10.1196/annals.1402.076 18056035

[B53] Robins J. C. Akeno N. Mukherjee A. Dalal R. R. Aronow B. J. Koopman P. (2005). Hypoxia induces chondrocyte-specific gene expression in mesenchymal cells in association with transcriptional activation of Sox9. *Bone* 37 313–322. 10.1016/j.bone.2005.04.040 16023419

[B54] Sánchez-Martín L. Estecha A. Samaniego R. Sánchez-Ramón S. Vega M. Á Sánchez-Mateos P. (2011). The chemokine CXCL12 regulates monocyte-macrophage differentiation and RUNX3 expression. *Blood* 117 88–97. 10.1182/blood-2009-12-258186 20930067

[B55] Schantz J. T. Chim H. Whiteman M. (2007). Cell guidance in tissue engineering: SDF-1 mediates site-directed homing of mesenchymal stem cells within three-dimensional polycaprolactone scaffolds. *Tissue Eng.* 13 2615–2624. 10.1089/ten.2006.0438 17961003

[B56] Schmittgen T. D. Livak K. J. (2008). Analyzing real-time PCR data by the comparative C(T) method. *Nat. Protoc.* 3 1101–1108. 10.1038/nprot.2008.73 18546601

[B57] Semenza G. L. (2001). HIF-1 and mechanisms of hypoxia sensing. *Curr. Opin. Cell Biol.* 13 167–171. 10.1016/s0955-0674(00)00194-0 11248550

[B58] Solchaga L. A. Penick K. Goldberg V. M. Caplan A. I. Welter J. F. (2010). Fibroblast growth factor-2 enhances proliferation and delays loss of chondrogenic potential in human adult bone-marrow-derived mesenchymal stem cells. *Tissue Eng. Part A* 16 1009–1019. 10.1089/ten.tea.2009.0100 19842915 PMC2862658

[B59] Tuli R. Tuli S. Nandi S. Huang X. Manner P. A. Hozack W. J. (2003). Transforming Growth Factor-β-mediated Chondrogenesis of Human Mesenchymal Progenitor Cells Involves N-cadherin and Mitogen-activated Protein Kinase and Wnt Signaling Cross-talk. *J. Biol. Chem.* 278 41227–41236. 10.1074/jbc.m305312200 12893825

[B60] van der Kraan P. M. van den Berg W. B. (2012). Chondrocyte hypertrophy and osteoarthritis: role in initiation and progression of cartilage degeneration? *Osteoarthritis Cartilage* 20 223–232. 10.1016/j.joca.2011.12.003 22178514

[B61] van Vlimmeren M. A. Driessen-Mol A. van den Broek M. Bouten C. V. Baaijens F. P. (2010). Controlling matrix formation and cross-linking by hypoxia in cardiovascular tissue engineering. *J. Appl. Physiol.* 109 1483–1491. 10.1152/japplphysiol.00571.2010 20847132

[B62] Wang D. W. Fermor B. Gimble J. M. Awad H. A. Guilak F. (2005). Influence of oxygen on the proliferation and metabolism of adipose derived adult stem cells. *J. Cell Physiol.* 204 184–191. 10.1002/jcp.20324 15754341

[B63] Weiss W. M. Mulet-Sierra A. Kunze M. Jomha N. M. Adesida A. B. (2017). Coculture of meniscus cells and mesenchymal stem cells in simulated microgravity. *npj Microgravity* 3:28. 10.1038/s41526-017-0032-x PMC5681589 29147680

[B64] Wickham M. Q. Erickson G. R. Gimble J. M. Vail T. P. Guilak F. (2003). Multipotent stromal cells derived from the infrapatellar fat pad of the knee. *Clin. Orthop. Relat. Res.* 412 196–212. 10.1097/01.blo.0000072467.53786.ca 12838072

[B65] Xu Y. Malladi P. Chiou M. Bekerman E. Giaccia A. J. Longaker M. T. (2007). In vitro expansion of adipose-derived adult stromal cells in hypoxia enhances early chondrogenesis. *Tissue Eng.* 13 2981–2993. 10.1089/ten.2007.0050 17916040

[B66] Zuk P. A. Zhu M. Ashjian P. De Ugarte D. A. Huang J. I. Mizuno H. (2002). Human adipose tissue is a source of multipotent stem cells. *Mol. Biol. Cell* 13 4279–4295. 12475952 10.1091/mbc.E02-02-0105 PMC138633

[B67] Zuk P. A. Zhu M. Mizuno H. Huang J. Futrell J. W. Katz A. J. (2001). Multilineage cells from human adipose tissue: implications for cell-based therapies. *Tissue Eng.* 7 211–228. 10.1089/107632701300062859 11304456

