# Supplementary material for: Inability of Low Oxygen Tension to Induce Chondrogenesis in Human Infrapatellar Fat Pad Mesenchymal Stem Cells
Source: Front Cell Dev Biol. 2021 Jul 26;9:703038. doi: 10.3389/fcell.2021.703038 (PMC8350173; doi:10.3389/fcell.2021.703038)
Supplement: Supplementary file 1 [file Table_1.DOCX]

**Table S1** Primer sequences for qPCR assay

| **Primer** | **Forward** | **Reverse** |
| --- | --- | --- |
| *β-Actin* | 5’-AAGCCACCCCACTTCTCTCTAA-3’ | 5’-AATGCTATCACCTCCCCTGTGT-3’ |
| *B2M* | 5’-TGCTGTCTCCATGTTTGATGTATCT-3’ | 5’-TCTCTGCTCCCCACCTCTAAGT-3’ |
| *YWHAZ* | 5’-TCTGTCTTGTCACCAACCATTCTT-3’ | 5’-TCATGCGGCCTTTTTCCA-3’ |
| *ACAN* | 5’-AGGGCGAGTGGAATGATGTT-3’ | 5’-GGTGGCTGTGCCCTTTTTAC-3’ |
| *COL1A2* | 5’- GCTACCCAACTTGCCTTCATG-3’ | 5’-GCAGTGGTAGGTGATGTTCTGAGA-3’ |
| *COL2A1* | 5’-CTGCAAAATAAAATCTCGGTGTTCT-3’ | 5’-GGGCATTTGACTCACACCAGT-3’ |
| *COL10A1* | 5’-GAAGTTATAATTTACACTGAGGGTTTCAAA-3’ | 5’-GAGGCACAGCTTAAAAGTTTTAAACA-3’ |
| *SOX9* | 5’-CTTTGGTTTGTGTTCGTGTTTTG-3’ | 5’-AGAGAAAGAAAAAGGGAAAGGTAAGTTT-3’ |
| *GREM1* | 5’-CATGTGACGGAGCGCAAATA-3’ | 5’-GCTTAAGCGGCTGGGTTTT-3’ |
| *TGFβ1* | 5’-GGGAAATTGAGGGCTTTCG-3’ | 5’-AGTGTGTTATCCCTGCTGTCACA-3’ |
| **Primer** | **Forward** | **Reverse** |
| *TGFβ2* | 5’-CGAGAGGAGCGACGAAGAGT-3’ | 5’-AGGGCGGCATGTCTATTTTG-3’ |
| *TGFβ3* | 5’-CTGGCCCTGCTGAACTTTG-3’ | 5’-AAGGTGGTGCAAGTGGACAGA-3’ |
| *IHH* | 5’-CCTTGTCAGCCGTGAGGCCG-3’ | 5’-GCTGCCGGCTCCGTGTGATT-3’ |
| *RUNX2* | 5’-GGAGTGGACGAGGCAAGAGTTT-3’ | 5’-AGCTTCTGTCTGTGCCTTCTGG-3’ |
| *ALPL* | 5’-CCTGGCAGGGCTCACACT-3’ | 5’-AAACAGGAGAGTCGCTTCAGAGA-3’ |
| *VEGF* | 5’-GCACGGTCCCTCTTGGAA-3’ | 5’-CGGTGATTTAGCAGCAAGAAAA-3’ |
| *P4Hα1* | 5’-GCAGGGTGGTAATATTGGCATT-3’ | 5’-AAATCAATTCCCTCATCACTGAAAG-3’ |
| *LOX* | 5’-AGGCCACAAAGCAAGTTTCTG-3’ | 5’-AAATCGCCTGTGGTAGCCATA-3’ |
