# Supplementary material for: Inability of Low Oxygen Tension to Induce Chondrogenesis in Human Infrapatellar Fat Pad Mesenchymal Stem Cells
Source: Front Cell Dev Biol. 2021 Jul 26;9:703038. doi: 10.3389/fcell.2021.703038 (PMC8350173; doi:10.3389/fcell.2021.703038)

**Supplementary Figure 1**.

Relative fluorescence unit (RFU) of images captured for Figure 7C using ImageJ. Image J (version 1.53e, NIH, USA) was used to measure the total area of each image with detected types I and II collagen fluorescence and to count the total number of nucleated cells indicated by 4',6-Diamidine-2'-phenylindole (DAPI) staining. The ratio of these values for each image was computed and plotted as shown for comparison between groups.


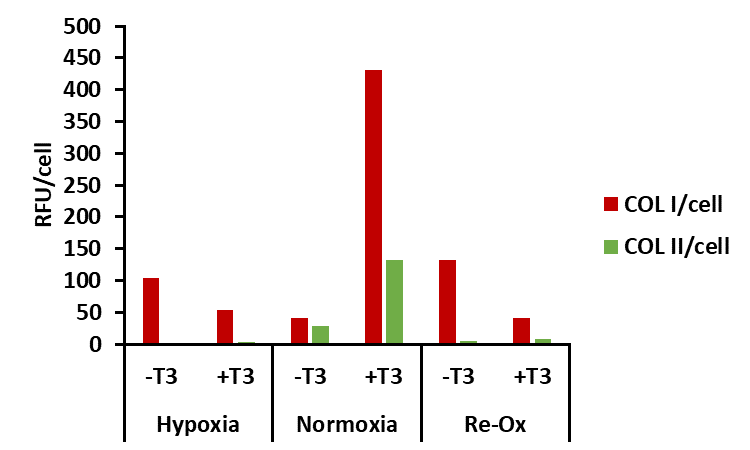

Supplement: Supplementary file 2 [file Data_Sheet_1.docx]
